# Nutritional Strategies for Childhood Obesity Prevention

**DOI:** 10.3390/life11060532

**Published:** 2021-06-08

**Authors:** Elena Fornari, Marco Brusati, Claudio Maffeis

**Affiliations:** 1 Department of Surgery, Dentistry, Pediatrics and Gynecology, Section of Pediatric Diabetes and Metabolism, University and Azienda Ospedaliera Universitaria Integrata of Verona, 37126 Verona, Italy; claudio.maffeis@univr.it; 2 Department of Medical and Surgical Sciences, University of Bologna, 40126 Bologna, Italy; marco.brusati@omceobs.it

**Keywords:** overweight, obesity, childhood, prevention, nutrition, diet, lifestyle

## Abstract

Background: Reducing the spread of obesity represents a challenge for clinicians in which obesity prevention plays a key role in achieving this purpose. The aim of this review is to analyze the nutritional interventions that can be implemented to prevent childhood obesity. Methods: Searching PubMed and Cochrane Library between 2019 and 2021. Further searching with no date range for articles selected for their specific relevance in the pediatric area or for their scientific relevance. A total of 871 articles were identified and 90 were included. Results: We organized the results of the selected articles into age groups, and according to the subjects targeted for interventions or to the site of interventions, reserving an in-depth analysis on specific nutritional aspects. Promotion of breastfeeding, reduction of protein content of formulated milks, and diet of the first 12–24 months, involving family and schools in interventions that promote physical activity and healthy diet, are promising strategies for reduction of the risk of obesity. To increase the efficacy of interventions, a multidimensional approach is crucial. Conclusions: A multidimensional approach, which takes into consideration different areas of intervention, is pivotal for childhood obesity prevention. Integrated programs involving several components (nutrition and physical activity at first) at different levels (individual, family, school, and institutional) are crucial.

## 1. Introduction

Overweight and obesity in childhood are growing worldwide, affecting not only the countries with the highest income but also the poorest ones [1,2]. The high prevalence of the disease and its persistence and consequent relationships with chronic diseases in adulthood make it essential to identify strategies to prevent its onset [2,3,4].

In 2016, it was estimated that there are 42 million children under the age of 5 with overweight or obesity and 50 million girls and 74 million boys aged 5–19 with obesity worldwide [5,6]. In Europe, the data collected in 2015–2017 by the World Health Organization in the context of the COSI (Childhood Obesity Surveillance Initiative) [7], which involved 35 nations, found a prevalence for overweight of 9–43% for boys and 5–43% for girls of 6–9 years; the prevalence for obesity was 2–21% among boys and 1–19% among girls. The European data show a north–south gradient with a higher prevalence in the nations of southern Europe [7]. Regarding severe obesity, rates are between 1.0% in Swedish and Moldovan children (95% CI 0.7–1.3 and 0.7–1.5, respectively) and 5.5% (95% CI 4.9–6.1) in Maltese children [8].

The excess weight that underlies overweight and obesity recognizes its cause in an imbalance between energy intake and energy expenditure, leading to a prolonged positive energy balance [1]. More than 600 genes have been associated with the risk of developing obesity [9]. However, in most cases, obesity does not have a major genetic cause but is the result of an interaction between genes and environment, which affects energy balance and metabolism [10]. In fact, in more the 95% of cases, obesity is the consequence of the inability of the genetically predisposed individual to adapt his/her behavior to the obesogenic environmental pressure [9]. The environmental exposition, i.e., food availability, cultural traditions and social behavior, area of residency, parents and caregiver behaviors, sport facilities availability since the fetal period, affects the risk of obesity through epigenetic regulation mechanisms throughout the course of life [10,11,12,13].

Unfortunately, the treatment of obesity is difficult and does not offer encouraging results in adults and also children [14,15]. On the contrary, prevention could represent an effective strategy [16]. There is common agreement that obesity prevention in children and adolescents is the first-line and high-priority approach to reduce obesity and, consequently, cardiovascular risk [2,17,18]. Prevention of childhood obesity must be directed at promoting behavioral changes from early life (pregnancy, infancy, and early childhood) to adolescence and young adult age, involving family, schools, society, media, and policy makers [2,16,19]. Developing effective intervention strategies and reducing long-term health costs associated with obesity is a major challenge for physicians [14].

The aim of this review is to report an update of the main nutritional interventions that can be implemented to prevent and contain the growing phenomenon of overweight and obesity in childhood. The analysis of the different areas of intervention from different points of view (age range, targets, settings), responds to the hypothesis that the multidimensional approach increases the effectiveness of interventions.

## 2. Materials and Methods

A narrative approach was applied in this review, aimed at describing the characteristics of the most successful nutritional interventions in preventing childhood obesity. Systematic reviews, meta-analyses, and consensus statements (with respect to individual trials) were taken into consideration.

We conducted a search in PubMed and in the Cochrane Library between July 2019 (date of publication of the Cochrane review on “Interventions for preventing obesity in children” [20]) and January 2021. Other filters applied: humans, English language, birth-18 years. Then we performed a further search with no date range, adding the name of journals as a limit, which were selected for their scientific relevance (i.e., *Lancet*, *New England Journal of Medicine*, *JAMA*) or for their specific relevance in the pediatric area (i.e., *JAMA Pediatrics*, *Obesity Review*, *American Journal of Clinical Nutrition*), to identify any publications that had escaped the first search. We mainly selected publications in the past 5 years but did not exclude commonly referenced and highly regarded older publications. Finally, we carried out an analysis of the bibliography of the most relevant studies to identify additional papers of interest. We used a combination of the following keywords: (“obes*” OR “overweight”) AND “prevent*” AND (“diet*” OR “nutrition*”) AND (“child*” OR “adolescen*” OR “pediatr*”). Inclusion criteria: review, systematic review, meta-analysis, randomized controlled trials and clinical trials targeting childhood obesity prevention and conducted in children aged 0–18 years. Studies targeting children with monogenic obesity or syndromic obesity or any pre-existing health conditions (e.g., diabetes and kidney disease) were excluded. Studies conducted among hospitalized children were also excluded from this review.

The final reference list was generated on the basis of originality and relevance to the broad scope of this review. In total, 871 articles were initially selected from which 90 were included in the review. The first two review authors (EF and MB) independently assessed potential study eligibility using predefined screening inclusion and exclusion criteria. Disagreements were resolved through discussion with the third review author (CM).

The identified studies were heterogeneous. We therefore organized the results into two age groups: “first 1000 days” and “preschool (2–6 years) and school age (6–18 years)” (Section 3.1 and Section 3.2, respectively). We then divided the first group according to the target of the intervention (parents, nutrition, etc.). In the second age group, we follow the subdivision by site of intervention (school, family, community, etc.). Finally, we reserved an in-depth analysis for some interventions on specific nutritional aspects (Section 3.3).

## 3. Results

### 3.1. Interventions in the “First 1000 Days”

#### 3.1.1. Interventions in Preconception Period and Pregnancy

Data about preconception interventions to prevent non-communicable diseases (NCD), including childhood obesity, are scarce, due also to the heterogeneity of the definition of “preconception” period [19]. Jacob et al., in 2019, analyzed the effect on intermediate outcomes related to the development of NCD [21]. Among the various approaches analyzed, the use of balanced protein-energy supplements has been shown to be successful (particularly in the group of malnourished mothers) in reducing the risk of SGA (small for gestational age) and LBW (low birth weight) births that are recognized risk factors for obesity [22]. A lower risk of gestational diabetes was identified among women with a higher level of physical activity [23]. Less weight gain during pregnancy was detected in an intervention group (nutrition and physical activity counseling or weight monitoring) compared to controls [24].

Interventions during pregnancy show poor efficacy in reducing childhood obesity, whereas positive effects can be found on maternal or neonatal conditions considered risk factors for childhood overweight/obesity [25,26]. Implementation of the First 1000 Days systematic interventions, starting in early pregnancy and lasting through the first 24 months of childhood, to prevent obesity among mother–child pairs was associated with modest reduction in excess gestational weight gain [25]. Balanced protein/energy supplementation, nutrition counseling, and exercise counseling were found to be useful in reducing these risk factors. In particular, energy/protein supplements to mothers “undernourished or nutritionally at risk” reduced the risk of SGA and LBW infants by 21% and 27%, respectively. A reduction in excessive weight gain during pregnancy was obtained with advice on low glycemic index foods (−24%) or with counseling on nutrition and physical activity (−16%). Dietary counseling reduced the risk of gestational diabetes by 46%, thus showing promise as it can act on a known overweight/obesity risk factors for the child [26].

#### 3.1.2. Interventions in the 0–2 Years Age Group

##### Breastfeeding

Breastfeeding is one of the most studied topics in terms of possible prevention of weight problems. A protective effect of breastfeeding on overweight and obesity was consistently noted [27], with a 13% reduction in the likelihood of excess weight gain during childhood and adulthood [28]. More discussed is the effect of the duration of exclusive breastfeeding on the same risks [27]. Regardless of whether breastfeeding is exclusive or not, it seems that a short duration compared to longer duration has less protective effects on the risk of overweight and obesity [27]. Finally, comparing exclusive vs. non-exclusive breastfeeding, the evidence is not conclusive regarding a greater protective effect of the first vs. the second [27]. The analyzed studies are heterogeneous on many aspects, such as methodology and clinical definitions, and some intervention studies, while reporting positive effects on breastfeeding, were not effective according to the anthropometric parameters of the child. Both the PROBIT study [29,30,31], which implemented the WHO/UNICEF “Baby-friendly Hospital Initiative” model to promote breastfeeding at a hospital level, and the study by Albernaz et al., which similarly focused on the promotion of breastfeeding, report positive effects in terms of the exclusivity and duration of breastfeeding without being effective on weight outcomes [32]. However, the studies that associate breastfeeding and growth parameters are exclusively observational studies (due to the unethical nature of a randomization of breast milk vs. formulated milk), so any confounding factors in the analysis cannot be completely excluded.

##### Characteristics of Formula Milk

Studies on the characteristics of formula milk in the first year of life in relation to the risk of overweight or obesity focus mainly on the protein content. Lower protein formulas are associated with lower weight and weight *z*-scores between 6 and 12 months, with lower BMI between 12 months and 6 years of age and with a lower risk of obesity at 6 years in the absence of conclusive data on body composition [33]. The considered studies differed widely in the protein content of milk with an overlap of concentrations between those defined as “lower protein formula” (1.1–2.1 g/100 mL) and the “higher protein formula” (1.5–3.2 g 100 mL). However, numerous authors confirm the same data. Hornell et al. [34] found that higher protein intake in the first two years of life is associated with higher BMI in pediatric age; Abrams et al. [35] similarly demonstrate that formulas with protein and energy content lower than those historically present in the United States are able to sustain adequate growth. Therefore, a reduction in the protein content of infant milks is promising but requires long-term evaluations to test its effectiveness over a longer period of time [33]. The current EFSA protein content limits for the formulated milks in the 0–12 month age range (1.8–2.5 g/100 kcal) [36] take these observations into consideration. The 2018 ESPGHAN Consensus on milks formulated for young children aged 1 to 3 years goes in the same direction, recommending a protein content toward the lower end of the permitted range for milks in the 6–12 month range [37].

The hydrolysis of milk proteins is another characteristic of formulated milks studied for its possible protective effects. The GINI study [38] found slower BMI growth in the first year of life in children who received an extensive casein hydrolysate, but later and up to the age of 10, there were no longer any differences in BMI between children who had taken extensive or partial hydrolysate vs. normal formulated milk or breastfed. A comparison between the extensive hydrolysate of milk proteins and normal formulated milk revealed lower growth (weight-for-length *z*-score) between 2.5 and 7.5 months for the children who took the hydrolysate [39]. Redsell et al., however, suggests caution in reading these data, reporting possible unfavorable characteristics of the use of hydrolyzed milks (flavor, allergies) [40].

About other interventions on formulated milks (supplementation with prebiotics or probiotics, LCPUFA, use of soy-based formulas), the evidence is currently insufficient to recommend their use as prevention strategies for overweight/obesity in children [27,41].

##### Complementary Feeding

Timing and protein intake are the two most studied aspects in relation to complementary feeding.

The ESPGHAN Consensus on complementary feeding (CF), reporting the association between the introduction of complementary foods before 4 months of age and subsequent greater adiposity, recommends that “complementary foods should not be introduced before 4 months of age but should not be delayed beyond 6 months “ [42]. Daniels et al., in their review conducted on 26 studies, conclude that “the introduction of solids before 4 months of age can result in a greater risk of obesity as a child” [43]. On the contrary, the 2019 EFSA Consensus [44] states that there is no clear association between the timing of CF and the risk of overweight or obesity, thus concluding that “the available data do not allow the determination of a single age for the introduction of CFs for infants living in Europe. The appropriate age range depends on the individual’s characteristics and development, even more so if the infant was born preterm. […] There is no convincing evidence that the introduction of CFs is associated with either adverse or beneficial health effects (except for infants at risk of iron depletion) at any age investigated in the included studies (<1 month to <6 months for earlier introduction)”.

About protein intake, the ESPGHAN Consensus [37] suggests not exceeding 15% of the total energy intake at the time of complementary feeding as a protective measure against subsequent overweight/obesity. Similar indications (range 8–12% and in any case <15%) are also reported in the latest edition of the Italian Nutrient Reference Intake Levels [45] in order to prevent weight problems.

##### Interventions on Parents

The numerous family-focused interventions to improve the growth parameters of children, with different characteristics and outcomes, make it difficult to draw general conclusions. Some RCTs published since the 1990s include interventions that involve parents or focus on parents [40,41,46]. Some of them started during pregnancy and have already been mentioned above. Many other studies concern only the postnatal period with great methodological differences, which made the comparison difficult. The study by Campell et al. followed up parents for 15 months with advice on nutrition, physical activity, and television exposure [47]. They reported, at 20 months of age, benefits related to the consumption of sweet snacks and in the time spent watching television, but they did not detect statistically significant differences in BMI. The Italian PROBIT study provided parents with information on protective behaviors with particular emphasis on responsive feeding: although a higher prevalence of protective eating behavior (breastfeeding on demand) was found at 3 months of age, there were no statistically significant differences in the prevalence of obesity at 2 years of age [48]. In the NOURISH study [49,50], the intervention began at 4–6 months of age of the child and was focused on healthy feeding and growth patterns. At 14 months of age in the control group, higher BMI *z*-scores were revealed, and the mothers more often used “non-responsive feeding practices”. However, at 2 years of age, while the mothers in the intervention group more often used responsive feeding, there were no longer statistically significant differences in the BMI *z*-score or in the prevalence of overweight/obesity.

In the SLIMTIME study [51], parents were educated to recognize the child’s hunger and satiety signals and other sources of possible distress: at 1 year of age, children whose parents had received both interventions had significantly lower weight-for-length percentiles than other subjects of the study. The American Heart Association, in a recent publication on this topic [52], mentions among the characteristics capable of supporting good “eating self-regulation” and a low risk of obesity, the parent’s responsiveness to the child’s hunger and satiety signals. It is emphasized to focus on the structured environment that the parent has to create (rules, limits, selective availability of food, role modeling), suggesting to effort the education of parents on these aspects as a strategy to reduce obesity and cardiometabolic risk throughout life.

### 3.2. Interventions in Preschool and School Age

#### 3.2.1. Preschool Setting

In a review conducted in 2015 by Wang et al., three RCTs evaluating nutrition and physical activity combined intervention for obesity prevention on preschool children were reported, with heterogeneous results [53]. The evidence of efficacy was low-grade due to the moderate risk of bias of the studies and the inconsistency of the results.

Bleich et al. analyzed 5 RCTs and 1 quasi-experimental study aimed to prevent childhood overweight and obesity, with a main setting in preschool education [54]. All five RCTs included the family as a secondary area of intervention. Three studies reported positive results: the first two exclusively with physical activity [55,56], the third using a multicomponent intervention [57]. The positive results consisted of a reduction in BMI in children aged 4–5 years in the first two studies, while the third found a lower percentile increase in BMI percentile and a greater consumption of fruit/vegetables in the subjects who received the intervention compared to controls. Two other trials with the proposal to assess the impact of a preschool-based obesity prevention program, focused on both physical activity and nutrition, report no difference in results between the intervention and control groups [58,59]. Zhou and colleagues reported data from a preschool intervention with a family and community involvement component and found positive effects on body composition in terms of fat and lean mass at 12 months of age of follow up, without detecting significant differences in BMI and BMI *z*-score [60].

#### 3.2.2. School Setting

School-based interventions mostly included elementary and middle school children and reported efficacy of these interventions with at least moderate evidence: about half of the studies analyzed in the review by Wang et al. report statistically significant positive effects on at least some measures of adiposity [53]. Interventions with a primary setting at school but with a component present at home had the highest proportion of studies with favorable outcomes. In the programs that included only the school setting, there was moderate evidence of a preventive effect against obesity in favor of interventions on nutrition or physical activity. In the programs involving school and home setting, the evidence of a preventive effect on obesity was insufficient for diet alone, while it was high for interventions on physical activity only and moderate for the combination of diet and physical activity. Furthermore, the meta-analysis of the school RCTs showed a difference in BMI *z*-score of −0.05 (95% CI: −0.10, −0.01) and BMI of −0.30 kg/m^2^ (95% CI: −0.45, −0.15). Finally, with regard to the school and community setting, the effectiveness of diet and physical activity in the prevention of obesity presented a moderate level of evidence [53].

Bleich et al. reported, out of 24 RCTs carried out in the school setting, 17 with favorable and statistically significant effects for at least one adiposity outcome [54]. Most of those with completely positive or mixed effects combined diet and physical activity interventions. Most RCTs with positive outcomes included the home environment as a secondary site of intervention. The difference in BMI between the intervention and control groups ranged from −0.33 to +0.05 kg/m^2^, with follow-up periods varying from 6 months to 6 years. The content of the interventions varied and included programs for strengthening and prolonging physical activity, implementation of courses on nutrition education, improvement of self-regulation and self-efficacy, changes in the environment and policies, etc. For the school setting, the authors also report 2 natural experiments and 15 quasi-experimental studies with conflicting results (6 null, 4 positive, 7 mixed) [54].

Bramante et al. reports data of only natural experiments [61]. Of the 33 selected studies, 8 had medium or low risk of bias and 6 of these took place in school. Five of these studies involved interventions on the food environment: 3 obtained positive results on BMI through a program focused on the availability of water to drink at school [62], through the improvement of the in- and extra-school food environment [63], and through modification of food and drink available at school [64]. The last one focused on physical activity and the built environment (walking school bus) showing a reduction in overweight/obesity [65].

#### 3.2.3. Home and Community Setting

Wang et al. reported interventions with a predominant home-based component and did not detect positive effects on outcomes related to diet adiposity [53]. Primary care interventions were not effective, whereas interventions on community and school setting, based on diet and physical activity, were able to prevent obesity (moderate evidence).

Community-level interventions show conflicting results and classify evidence in favor of community-based initiatives as weak [54,66]. The Cochrane review of 2019 reported the effectiveness of the interventions conducted both at home and at the community level [20], and community and home-based child obesity interventions seems to have lower costs per child and greater compliance, with similar impacts [67]. Approaches that modify the food environment (vending machines at school, installing water fountains, advertising) and facilitate physical activity, perhaps also achieved through the internet and social media, can be suitable for older children and adolescents [2,20]. Interventions on fast-food marketing or on subsidies for the poorer classes might also be useful [46].

Narzisi et al. also highlight that studies support the community interventions that involve layers of society surrounding children, such as children’s services, schools, and homes, supporting behavioral change and representing a useful approach to tackling childhood obesity [68].

### 3.3. Interventions on Specific Aspects of Dietary Intake

Healthy eating habits have a pivotal role on preventing overweight/obesity. Some aspects of nutritional interventions specific for certain ages have already been considered in the first part of this review (e.g., protein intake), while others which affect overall changes in eating habits, even regardless of age, are discussed below.

Two recent Cochrane reviews address the issue of fat intake and its effect on weight. The cohort studies show that there is a trend of increase in adiposity with the increase in total fat intake [69]. From the analysis of three RCTs (4–13 years old), the authors report that a counseling/education intervention towards a reduction in lipid intake (≤30% vs. >30% of total energy intake) effectively reduce lipid intake (total and saturated) and determine a reduction in BMI (−1.5 kg/m^2^, 95% CI: −2.45, −0.55; moderate quality of evidence). The review conducted in 2020 [70], due to the poor quality of the available evidence, fails to draw conclusions about the effectiveness of a law on total fat or saturated fat content in terms of reducing their consumption, energy intake, or prevention of overweight or obesity. As regards the intake of polyunsaturated fats (PUFA) Patro-Golab reports that the evidence deriving from different types of interventions from birth to the first years of life is not conclusive regarding the intake of PUFA and the subsequent risk of overweight or obesity or fat mass [27]. The INFAT study provided for supplementation with n3 LCPUFA and reduction in the intake of arachidonic acid during pregnancy and in the first 4 postnatal months but did not detect differences in terms of adiposity up to 12 months of age [71].

The evidence about the influence of energy intake in early childhood and subsequent growth is limited [27]. Indirect data on this aspect can be derived from responsive feeding studies. In the SLIMTIME study, the intervention was specifically aimed at empowering parents in recognizing child’s hunger and satiety signals and resulted in lower weight-for-length at one year of age [51]. On the other hand, strategies that are able to promote responsive feeding in early childhood do not necessarily determine positive results on weight parameters [48].

About sugar intake, the 2015 WHO document “Sugars intake for adults and children” affirms that there is moderate evidence “for an association between a reduction in the intake of free sugars and a reduction in body weight” and poor evidence “for an association between an increase in free sugar intake and an increase in body weight” [72]. Four of the reviews taken into consideration report a possible reduction in the intake of sugary drinks following the implementation of different types of interventions [27,40,46,61]. However, the evidence is contradictory regarding an association between the intake of sugary drinks in the first years of life and long-term overweight or obesity, also due to a likely confounding effect of other nutritional habits of the same subjects [27]. However, a reduction in the intake of sugary drinks, due to the association between their consumption and overweight/obesity, is likely to translate into benefits in terms of weight [73,74]. The recent Position Paper of the Global Federation of International Societies of Paediatric Gastroenterology, Hepatology and Nutrition (FISPGHAN) recommends the promotion of drinking water instead of sugar-containing beverages from early childhood [2]. The use of calorie-free sweeteners is still discussed in terms of their preventive or therapeutic effect on weight. The American Academy of Pediatrics’ 2019 Policy Statement states that they can reduce weight gain or promote small weight loss and, therefore, subjects with certain conditions such as obesity can benefit from them when used in place of caloric sweeteners [75]. However, the authors also warn against believing that they can lead to significant weight reductions when used as the only strategy and that many aspects remain to be clarified in terms of safety [75].

The healthy effects of fibers were recently analyzed by Reynholds et al., which evaluates 45 observational studies in children aged 1 to 19 years, identifying benefits on body weight, blood lipids, blood pressure, blood glucose associated with an increased intake of fiber with the diet and high-fiber foods, in the absence of adverse events [76]. Positive effects of fiber supplementation on reducing appetite and triglycerides absorption are also demonstrated after a single intake, in the postprandial phase in the obese children [77]. For the consumption of fruit and vegetables, Bramante et al. report positive results of interventions in the school environment in 2 out of 5 studies [61].

Finally, the Mediterranean diet is suggested as a protective eating style, not only in the latest guidelines of the Italian Society of Pediatric Endocrinology and Diabetology [2,78]. In the multitude of studies published on this topic [79], the Fernandez-Barres study evaluates the rate of adherence to the Mediterranean diet during pregnancy by relating it to the anthropometric parameters of offspring [80]. The authors identify a positive effect of adherence to the Mediterranean diet on the waist circumference in the absence of effects on the BMI z-score at 4 years of age. Pereira-da-Silva et al. highlights how there is an inverse relationship between adherence to the Mediterranean diet and the risk of overweight in children [81]. D’Innocenzo et al. analyze the relationship to the Mediterranean diet adherence and the increase in the incidence of overweight and obesity, considering also pediatric age [79].

Promotion of adequate nutritional habits and healthy lifestyle does not only recommend a balanced diet but also encourages the modification or maintenance of some correct lifestyle habits that, in turn, affect the eating habits of the whole family [2,17,78]. Food intake should be distributed in no more than 5 daily meals (three principal meals and no more than two snacks) and home consumption of meals should be promoted [2,17,78]. To encourage the daily intake of an adequate breakfast is part of the supportive actions suggested by pediatric guidelines in order to prevent overweight and obesity [17,78].

## 4. Discussion

The analyzed studies show marked heterogeneity. The subjects involved can be the parents of the subjects on whom the effects of the intervention are to be evaluated or the children themselves, evaluated at different ages (from the first months of life to 18 years). The interventions range from preschool and school settings to the family, the hospital environment, and the community. The identified studies also try to analyze all possible interventions capable of influencing the child body weight, thus investigating possible associations with the mother’s or child’s diet, behaviors of parents which can influence children food intake, impact of physical activity, changes in the environment in which the study subjects live, etc. The intensity of the interventions is also variable, so a physical activity program could include only education about the role of physical activity, up to daily sessions of specific intensity physical activity [20]. The duration of the intervention and follow-up frequently differs between studies. The duration of the follow-up is a critical point since obesity is a chronic disease. Only few studies continue beyond the active intervention period alone and, therefore, no conclusions can be drawn on medium- and long-term effects [41,53]. However, many of the studies led to changes in policies, environment, or staff training, which could therefore have effects persisting after the end of the study period [54]. Furthermore, the Cochrane review by Brown and colleagues in the conclusions states: “the behaviors related to nutrition and physical activity adopted as a child are prolonged during life” [20]. It is possible that the small changes identified in the short term can add up, giving long-term benefits on different aspects of health.

An important issue is the impact that the identified positive results could have at an individual level and at a community level. The advantages in terms of BMI and BMI z-score are often limited. Bleich reports a variation in BMI from −0.05 to −0.33 kg/m^2^ [54], Wang −0.30 kg/m^2^ [53], Brown a maximum of −1.53 kg/m^2^ [20], and Salam −0.41 kg/m^2^ [67]. For the BMI z-score, Wang reports a variation of −0.05 [53], Bramante of at least −0.15 [61], Brown a maximum of −0.20 [20], and Salam a maximum of −0.41 [67]. The authors express doubt about the impact of these relatively limited results, considering the global increase in BMI recorded in recent decades, and question their clinical significance [20,53,54]. Brown believes that they could translate into a significant effect at a population level if their effect could be sustained for years but the lack of long-term data cannot allow this to be concluded [20]. McCrabb et al. recently analyzed the difference in the effectiveness of obesity prevention/treatment interventions in the comparison between RCTs and the same interventions implemented in real life [82]. The effects on BMI and BMI z-score found in RCTs are significantly lower when the interventions are implemented in real life, making the impact of results even more uncertain. On the other hand, Redsell et al. point out that not stratifying the results by the level of risk of overweight/obesity in the sample may not evidence significant outcomes in low-risk individuals [40].

The studies analyzed show a paucity of information about the costs of the interventions and therefore on their cost-effectiveness. None of the studies reported by Wang et al. report cost estimations to achieve the observed effects [53], and in the Cochrane review less than 10% of the studies reported the costs [20]. Neither the first nor the second review reports cost-effectiveness estimation of the interventions. Salam et al. reports that only a few of the 359 studies on obesity prevention reported cost-effectiveness estimations which, however, were generally favorable [67].

Three studies report on possible adverse effects [20,53,67]. These could be “psychological”, e.g., the stigma deriving from the measurements and their interpretation or a sense of failure if the desired results were not obtained, or “physical”, such as trauma related to physical activity. In particular, the Cochrane review points out that only a small number of studies reported data on this aspect but that the reported did not detect any dangers to children [20].

Despite the heterogeneity of studies and results, some common remark can be found (Table 1).

Interventions on parents in the period from conception to the first 2 years of life are generally effective in terms of behavior change but do not always have an impact on growth or adiposity parameters [13,40,41]. Family-based interventions are the most effective on weight status when compared with approaches in other contexts (e.g., healthcare setting, community) [46].

From 2 years of life, the interventions that are most consistently effective in preventing obesity in children are those conducted in a school setting [53,54,61], in particular if they include a component carried out in the family environment [53,54]. This is even clearer, considering that children spend a significant part of the day in preschool and school settings, often consuming more than one meal there a day. Furthermore, the school already has a structural and conceptual infrastructure that includes the task of education and change of habits [67]. Family involvement also plays a pivotal role given the influence that the family and the domestic environment have on the health-related behaviors of children and adolescents [83,84], widely recognized by organizations dealing with the problem [5,78,85]. Partially in contrast with these two reflections are the conclusions of the Cochrane review, that does not identify effects deriving from interventions on diet and physical activity conducted in the preschool setting and, on the contrary, found efficacy in interventions conducted at home or in the community [20]. However, caution is necessary in comparing these data due to the different subdivision by age group between the revisions considered.

About the effectiveness of the different interventions analyzed in the age group from conception to 2 years, some strategies focused on nutrition appeared to be particularly promising. The promotion of breastfeeding and the reduction in the protein content of formulated milks and more generally of the diet in the first 12–24 months is another promising intervention for the reduction of the subsequent risk of overweight and obesity [27,28,33,37,40,41,42,86,87,88]. Although still partially discussed [44], the advice to avoid the introduction of complementary foods before 4 months can be considered useful for reducing the rates of overweight and obesity [27,42,85]. From 2 years of age, the studies are substantially in agreement in considering the approaches that combine interventions on nutrition and physical activity to be more effective [20,53,54,67]. However, an efficacy of approaches based on nutrition or physical activity individually is not excluded, depending on the age of the subjects and the contexts of application [20,53,54,61]. This result is perfectly consistent with the theoretical model of obesity as a result of an imbalance between the intake of energy with the diet and energy expenditure with physical activity: acting on both these aspects, more consistent results are obtained. The universal advice (from pregnancy to old age) to follow a healthy diet with particular reference to the Mediterranean diet model as a protective approach against overweight and obesity in childhood recurs, more or less explicitly, in many of the considered papers [27,40,41,61,80,81,86]. A reduction in the intake of sugary drinks is likely to translate into benefits in terms of weight, whereas the use of caloric sweeteners is still being discussed, both in terms of safety and effectiveness in reducing weight gain [73,74,75]. The intake of fruit, vegetables, and foods rich in fiber has positive effects on various health parameters (satiety, body weight regulation, glycemic control, blood lipids, intestinal microbiota modulation, etc.) [76,81,86]. Therefore, as Pereira-da-Silva concludes in his systematic review on the diet of preschool children, the interventions that determine an improvement in the intake rates of these foods should be implemented and “daily consumption of whole fresh fruit and vegetables should be promoted” [81].

The need for a multidimensional approach to obesity prevention in children, in order to increase the efficacy of interventions, is an aspect that recurs in many studies [2,5,13,67,68]. The WHO Commission on Ending Childhood Obesity suggests that part of the responsibility for the failure of interventions that influence behavior changes of the individual is precisely linked to the fact that they concern only the individual subject [5]. Since obesity is a multifactorial pathology, a multi-level and diversified approach according to the target is necessary. In the prenatal, infant, and preschool age, the training of parents and caregivers on issues such as healthy nutrition, physical activity, and sleep hygiene can have a significant impact [20]. In older children and adolescents, an approach that modifies the food environment (vending machines, water fountains, advertising) and facilitates physical activity may be more suitable [2,20]. At a higher level, prevention programs should imply the gradual remodeling of economic, agricultural, industrial, environmental, socio-educational, recreational and health policies, including those aimed at contrasting socio-economic and ethnic minorities’ inequalities [17,78,89]. Finally, a concept that summarizes the efforts in obesity prevention is that expressed by the Italian national program “Gaining health” [90]: “Making healthy choices easier and less easy harmful choices” [90], or even more recently by the American Institute of Medicine “Create food and beverage environments that ensure that healthy food and beverage options are the routine, easy choice” [85]. These claims mean that it is necessary to act at a policy level to make the healthier choice easier and immediate compared to the less healthy one. This would make the choice of these “protective” foods/drinks passive, against their active research which, on the contrary, would require effort and time and therefore would not be always easily practicable.

Our study has some limitations. First, the search performed focused on only two databases (PubMed and Cochrane Library) and, thus, may have missed some relevant publications. The search then performed manually should however have reduced this occurrence. Second, more emphasis has been placed on studies published since 2015 (in particular, 9 of the 14 reviews considered in the large part of the work base were published in 2019 or 2020). This choice aimed to find recent data for the conclusions drawn; on the other hand, it could have caused the loss of some relevant information published at an earlier time point. Third, some of the conclusions were based on observational studies, for which confounding factors in the results cannot therefore be excluded. Fourth, the fact of having performed a search for reviews, only evaluating the original studies from which they derive in some cases, has probably led to the inclusion of the same study in more than one review, even if the reviews often included different kinds of studies and different age groups. Finally, the use of BMI as the main outcome of many studies may not always be satisfactory as it may not show changes in body composition consisting, for example, of a reduction in fat mass associated with an increase in lean mass.

## 5. Conclusions

The prevention of childhood obesity still represents a major challenge for the scientific community. A multidimensional approach, which takes into consideration different areas of intervention, is crucial. Integrated programs involving several components (nutrition and physical activity at first), different levels (from the individual to the institutions, with school and family as main settings), and with a prolonged duration over time, are more effective, but long-term data are still scarce. More studies need to be conducted from the early stages of life and new success indicators in addition to BMI, such as more direct measurement of fat and lean mass, quality of life, and cost-effectiveness, should be included in future studies. Nevertheless, policy intervention, inducing the whole community to make healthier choices, could help to protect from the obesogenic environment. Pediatricians, who follow the child from birth, play a key role in prevention, and should intervene early to guarantee active family involvement associated with nutritional and lifestyle counseling.

## Figures and Tables

**Table 1:** Nutritional interventions that can contribute to prevent childhood obesity, classified by age groups.

Age Groups	Type of Intervention
Preconception interventions	Use balanced protein-energy supplements in malnourished mothers (to reduce the risk of SGA and LBW). Provide nutrition and physical activity counseling. Stop smoking (to reduce the risk of LBW).
Pregnancy	Use energy/protein supplements in mothers “undernourished or nutritionally at risk” (to reduce the risk of SGA and LBW). Stop smoking (to reduce the risk of LBW). Control maternal diabetes and GDM.
From birth to the first 2 years	Favor breastfeeding.
	Not introduce complementary feeding before 4 months of age.
	Recommend infant formula milks with appropriate protein content.
	Promote the active involvement of the family.
From the age of 2	Combine intervention on nutrition and physical activity. Promote the active involvement of the family. Involve the school setting in the interventions. Promote the consumption of a healthy diet, with particular reference to the Mediterranean diet. Increase fiber intake. Respect the RDI for lipids. Reduce the intake of sugary drinks.

List of abbreviations. SGA: small for gestational age; LBW: low birth weight; GDM: gestational diabetes mellitus; RDI recommended daily intake.

## Data Availability

Not applicable.
